# Areas of work‐life, psychological capital and emotional intelligence on compassion fatigue and compassion satisfaction among nurses: A cross‐sectional study

**DOI:** 10.1002/nop2.2098

**Published:** 2024-02-08

**Authors:** Stéphanie Maillet, Emily A. Read

**Affiliations:** ^1^ Faculty of business administration Université de Moncton Moncton New‐Brunswick Canada; ^2^ Faculty of Nursing University of New Brunswick Fredericton New Brunswick Canada

**Keywords:** areas of work‐life, compassion fatigue, compassion satisfaction, emotional intelligence, licensed practical nurses, psychological capital, Registered Nurses

## Abstract

**Aim:**

To examine the impact of six areas of work‐life, emotional intelligence and psychological capital on compassion fatigue and compassion satisfaction among Canadian Registered Nurses and licensed practical nurses.

**Design:**

A cross‐sectional survey study.

**Methods:**

A convenience sample of 296 Registered Nurses and 110 licensed practical nurses answered a self‐administered questionnaire measuring areas of work‐life, psychological capital, emotional intelligence, compassion satisfaction and compassion fatigue in September 2019. The association between variables were analysed with descriptive and correlational analyses, while the hypothesized models were tested using multiple regression analyses.

**Results:**

This study identified several areas of work‐life and intrapersonal resources that impacted compassion satisfaction and compassion fatigue. Among Registered Nurses, compassion satisfaction was predicted by psychological capital, rewards, values and workload. Compassion fatigue was predicted by psychological capital, workload, control and community. Among licensed practical nurses, compassion satisfaction was predicted by psychological capital and emotional intelligence. Compassion fatigue was predicted by workload and psychological capital. Study results also revealed significant differences in Registered Nurses' and licensed practical nurses' perceptions of workload, rewards and fairness at work, and both compassion satisfaction and compassion fatigue. Registered Nurses perceived their workload to be more manageable and perceived greater rewards and greater fairness at work than licensed practical nurses. Compassion fatigue was higher for Registered Nurses than licensed practical nurses, while compassion satisfaction was higher for licensed practical nurses than Registered Nurses. Future studies should investigate the nature and span of these differences to suggest relevant strategies to mitigate compassion fatigue and promote compassion satisfaction for each of these nursing roles.

**Conclusion:**

The results of this study underscore the need to create nursing work environments that foster a manageable workload and positive social relationships, where nurses have professional autonomy, decision‐making capacities and access to adequate resources to do their job effectively. The nursing work environment should also provide recognition of nurses' contributions and an alignment between personal and organizational values. Investments in the development and improvement of nurses' psychological capital and emotional intelligence should be prioritized since they are malleable and impactful intrapersonal resources on compassion satisfaction and compassion fatigue.

**Reporting method:**

This study adhered to the STROBE guidelines.

**Public contribution:**

A total of 406 nurses were involved in this study by answering a self‐administered study survey.

## 1 INTRODUCTION

As the largest workforce in Canadian healthcare (Canadian Institute for Health Information, 2021), nurses must provide compassionate care to patients and their families in fast‐paced and demanding work environments (Yildrim et al., 2021). The chronic physical, emotional and psychological toll of providing quality care in complex and high‐pressure environments can become a “cost of caring” for nurses. This is due, in part, to the profession's relational nature, which can not only promote nurses' compassionate connections with patients but also deplete their emotional resources due to the inherent stress of nursing practice (Maddigan et al., 2023; Zhang et al., 2018). Such an unrelenting and emotional work environment can lead to increased compassion fatigue and reduced compassion satisfaction, which contributes to a panoply of negative outcomes for nurses, patients and healthcare organizations (Al‐Marri et al., 2020; Yu et al., 2021). According to Barzgaran et al. (2019), the onset of compassion fatigue and reduction of compassion satisfaction can disrupt nurses' performance and, ultimately, reduce the quality and outcomes of patient care delivery. If progress is to be made on reducing nurses' compassion fatigue and increasing compassion satisfaction, it is crucial to identify key antecedents that impact their prevalence. Previous studies have shown that nurses' perception of their work environment and their ability to cope with professional stressors contribute to their compassion fatigue and compassion satisfaction. However, few studies have investigated these relationships among licensed practical nurses (LPNs), let alone in comparison to Registered Nurses (RNs). This study examines organizational (areas of work‐life) and personal (emotional intelligence and psychological capital) predictors of compassion fatigue and compassion satisfaction among RNs and LPNs.

## 2 BACKGROUND

Despite frequent exposure to the various stressors that characterize the nursing profession, such as workforce shortages, excessive workloads and time constraints, nurses continue to experience a sense of personal gratification from providing care. They value their work because it makes a difference in people's lives (Peters, 2018; Sinclair et al., 2016; Wang et al., 2020; Xie et al., 2021). In the context of caregiving, these positive experiences are often referred to as “compassion satisfaction” (Stamm, 2002). Compassion satisfaction has been associated with high‐quality and comprehensive care, increased patient satisfaction, improved patient outcomes and enhanced well‐being among nurses (Gelkop et al., 2022; Sinclair et al., 2016). However, a nurse's capacity to provide compassionate care is limited by the environment in which they must provide it (Towey‐Swift & Whittington, 2021). Evidence has demonstrated that overburdened work environments can increase nurses' vulnerability to compassion fatigue (Sinclair et al., 2017; Xie et al., 2021). Compassion fatigue represents a negative impact of nurses' work‐related stress, and decreases care capacity (Maddigan et al., 2023; Stamm, 2010; Yesil & Polat, 2023).

Peters (2018) defined compassion fatigue as “a preventable state of holistic exhaustion that manifests as a physical decline in energy and endurance, an emotional decline in empathetic ability and emotional exhaustion, and a spiritual decline as one feels hopeless or helpless to recover those results from chronic exposure to others' suffering, compassion, high stress exposure, and high occupational use of self in the absence of boundary setting and self‐care measures” (p. 470). Nurses who report experiencing compassion fatigue often distance themselves and withdraw from emotional connections with patients and families as a coping mechanism, thereby disrupting compassionate care behaviours (Nolte et al., 2017). Past research has also linked nurses' compassion fatigue to a panoply of physical, psychological, social and spiritual consequences, such as insomnia, depression, poor judgement, strained nurse–patient relationships, increased medical errors and disputes, reduced productivity, high staff turnover, lack of spiritual awareness and reduced ability to feel empathy (Wijdenes et al., 2019; Xie et al., 2021).

Since compassion is an essential attribute of high‐quality nursing (Nolte et al., 2017), and given the positive influence of compassion satisfaction and the negative influence of compassion fatigue on outcomes for patients, nurses and healthcare organizations, it is fundamental to understand the personal and organizational factors that contribute to these phenomena. Given the significant impact of the work environment on nurses' job‐related outcomes, we suggest that nurses who feel a strong alignment between their desired and actual working conditions are likely to experience higher levels of compassion satisfaction and lower levels of compassion fatigue. We also believe that nurses can mobilize personal resources to mitigate the impact of stressful working environments. In line with previous research, we argue that emotional intelligence and psychological capital may act as additional buffers to prevent compassion fatigue while fostering compassion satisfaction (Bao & Taliaferro, 2015; Peters, 2018).

## 3 THE STUDY

This research tested hypothesized models examining the impact of person‐job fit with six areas of work‐life, emotional intelligence and psychological capital on compassion satisfaction (model a) and compassion fatigue (model b) among RNs and LPNs.

### 3.1 Areas of Work‐Life

The Areas of Work‐Life model (Leiter & Maslach, 2004; Maslach & Leiter, 1997) identifies six key areas of work‐life that influence nurses' perception of the match between their expectations and their actual practice environment (also known as person‐job fit). The six areas of work‐life include workload (physical and emotional demands under time and resource constraints), control (opportunities and ability to make autonomous decisions about one's work), reward (recognition for one's contributions), community (quality of social relationships), fairness (consistent and impartial support and decision‐making processes) and values congruence (match between personal and organizational values) (Boamah & Laschinger, 2016; Leiter & Maslach, 2004; Maslach & Leiter, 1997; Read & Laschinger, 2013). Previous research has shown poor person‐job fit between these six areas of work‐life to be associated with emotional exhaustion, turnover intentions, burnout and poor work engagement among nurses (Bamford et al., 2013; Boamah & Laschinger, 2016; Brom et al., 2015; Leiter & Maslach, 2009). Yet, despite these relationships with related concepts, previous studies have not examined the impact of person‐job fit with these six areas of work‐life on compassion satisfaction and compassion fatigue. Therefore, a deeper understanding of how each of these six areas of work‐life impacts compassion satisfaction and compassion fatigue may serve as a means of improving nurses' work environments and, ultimately, quality of care for patients.

### 3.2 Emotional intelligence

Human relationships and emotions are an integral part of quality nursing care (Dugué et al., 2021; Nightingale et al., 2018). Emotional intelligence is thus considered an essential ability that helps nurses deliver effective nursing interventions and provide compassionate patient care (Castelino & Mendonca, 2021; Lu & Shorey, 2021). There is a growing body of evidence suggesting that nurses with a sufficient level of emotional intelligence are better able to meet the demands of their work and attain better patient outcomes and patient satisfaction (Adams & Iseler, 2014; Gelkop et al., 2022; Lu & Shorey, 2021). Thus, we propose that nurses with higher levels of emotional intelligence may be better equipped to deal with the emotional demands their job requires and, consequently, more likely to experience compassion satisfaction and less likely to experience compassion fatigue.

### 3.3 Psychological capital

Psychological capital is an intrapersonal resource comprised of hope, efficacy, resilience, and optimism, or the “HERO” within (Luthans et al., 2004; Yildrim et al., 2021). When dealing with their stressful and emotionally difficult work demands, it may be helpful for nurses to mobilize their “HERO” resources to recover more quickly and thereby reduce compassion fatigue and foster compassion satisfaction (Yildrim et al., 2021). Encouraging results from a study among acute care nurses conducted by Bao and Taliaferro (2015) demonstrated that psychological capital is negatively associated with burnout and secondary traumatic stress, which are two well‐known dimensions of compassion fatigue (Stamm, 2010), while it is positively correlated with compassion satisfaction. Our study builds on this work by testing the impact of psychological capital on compassion satisfaction and compassion fatigue among RNs and LPNs.

### 3.4 Significance

The relationships between areas of work‐life, emotional intelligence, psychological capital and both compassion fatigue and compassion satisfaction have received little attention in prior literature. Therefore, this research aims to bridge this empirical gap by examining the relationships between these variables among RNs and LPNS.

In addition, despite the considerable amount of research conducted on the RNs' work environment, there is a sizeable gap in the current nursing literature examining the work experiences of other nursing roles, particularly LPNs, which make up a significant part of the Canadian nursing workforce (Phillips et al., 2021). Indeed, it is unclear whether RNs and LPNs have similar antecedents of compassion fatigue and compassion satisfaction. In Canada, both RNs and LPNs are provincially regulated nursing professions that draw from the same clinical knowledge base, work collaboratively and share responsibility for coordinating and delivering clinical patient care (Canadian Institute for Health Information, 2021; Montayre & Montayre, 2017; Phillips et al., 2022; Squires et al., 2019; Thompson et al., 2021). However, due to greater education requirements, RNs' scope of practice is wider than LPNs and requires greater leadership, critical thinking and clinical judgement (Phillips et al., 2021; Thompson et al., 2021). Considering the different scopes of practice of RNs and LPNs, it is important to examine how both groups of nurses experience their work. To date, no studies have examined compassion satisfaction or compassion fatigue among LPNs. Therefore, this study addresses an important gap in knowledge about this occupational group.

### 3.5 Aims and objectives

**FIGURE 1:**
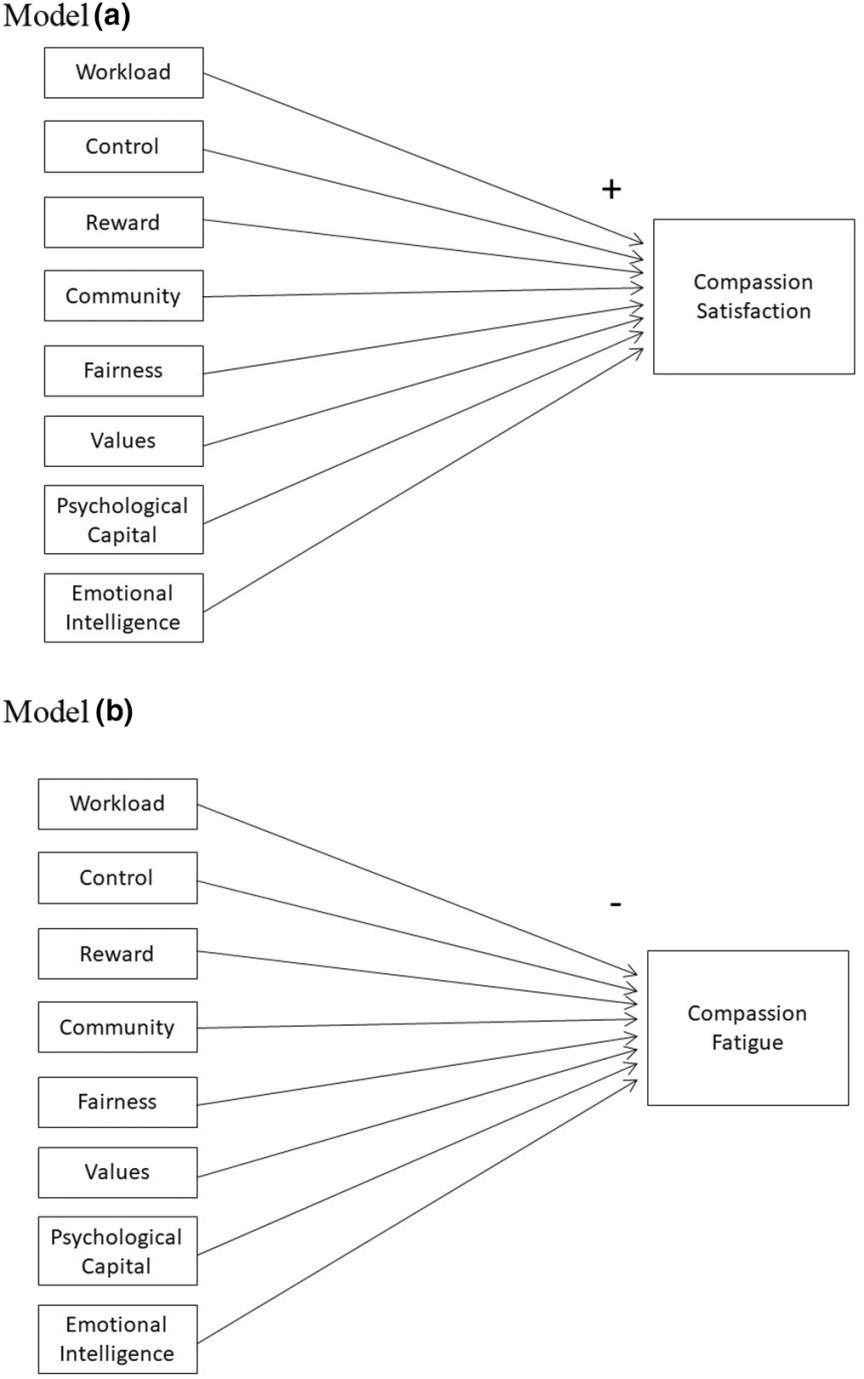
Hypothesized models. Model a: Compassion satisfaction on areas of work‐life, emotional intelligence and psychological capital. Model b: Compassion fatigue on areas of work‐life, emotional intelligence and psychological capital.

This study's main objective was to examine the impact of six areas of work‐life, emotional intelligence and psychological capital on compassion fatigue (Figure 1a – model a) and compassion satisfaction (Figure 1b – model b) among a sample of Canadian RNs (*n* = 296) and LPNs (*n* = 110). First, we hypothesized that person‐job fit between the six areas of work‐life, emotional intelligence and psychological capital would be positively associated with compassion satisfaction, such that higher levels of each predictor variable would result in greater compassion satisfaction. Second, we hypothesized that person‐job fit between the six areas of work‐life, emotional intelligence and psychological capital would be negatively associated with compassion fatigue, such that higher levels of each predictor variable would result in lower compassion fatigue. This study also aimed to examine the differences of these hypothesized models between RNs and LPNs.

## 4 METHODS

### 4.1 Design

A cross‐sectional survey of RNs and LPNs was conducted.

### 4.2 Instruments

Data were collected using a self‐report survey comprised of demographic questions and previously validated questionnaires which have demonstrated acceptable validity and reliability in previous research. Permission was obtained to use the study questionnaires. Participants were asked to indicate their gender, age and years as a nurse. Each of these demographic variables were categorical.

The Areas of Work‐life Questionnaire (Leiter & Maslach, 2004) was used to assess employees' perceptions of person‐job fit with six areas of their work‐life: workload (5 items), control (4 items), reward (4 items), community (5 items), fairness (6 items), and values congruence (4 items). Each item is rated on a Likert scale ranging from 1 = strongly disagree to 5 = strongly agree. Eight items are reverse‐coded. Subscale scores were calculated by taking an average of the items within each subscale. Previous research has supported the construct validity and internal consistency of this questionnaire (Leiter & Maslach, 2004). Subscale reliabilities ranged from 0.59 to 0.82 (RNs) and 0.69 to 0.81 (LPNs) in the current study. Questionnaires with a Cronbach's alpha of 0.60 or above are considered to have adequate internal consistency (Cronbach, 1951; Hajjar, 2018).

The Psychological Capital Questionnaire (developed and validated by Luthans et al., 2007) was used to assess nurses' perceived psychological capital. This 24‐item questionnaire is comprised of four subscales: hope (6 items), resiliency (6 items), optimism (6 items), and efficacy (6 items). Three items are reverse‐coded. Subscale scores were calculated by taking an average of items within each subscale. Total scores were calculated by taking an average of the four subscale scores. Cronbach's alpha for the total scale was 0.92 for both RNs and LPNs, with subscale reliabilities ranging from 0.71 to 0.88 (RNs) and 0.70 to 0.91 (LPNs).

Emotional Intelligence was assessed using 29 items from the Schutte Self‐Report Emotional Intelligence test (SSEIT) (Schutte et al., 1998). Items are rated on a Likert scale from 1 = Strongly Disagree to 5 = Strongly Agree. Two items are reverse coded. The 6‐factor measurement structure supported by Jonker and Vosloo (2008) was used in this study: positive affect (7 items), emotion—others (7 items), happy emotions (4 items), emotions—own (4 items), non‐verbal emotions (3 items) and emotional management (4 items). Subscale scores were calculated by taking the average of items within each subscale. Total EI was then calculated by taking the average of the six subscale scores. In the current study, overall Cronbach's alpha was 0.87 for RNs and 0.86 for LPNs.

The revised Professional Quality of Life (ProQOL‐21) item and scoring approach (Heritage et al., 2018) was used to assess compassion fatigue (11 items) and compassion satisfaction (10 items). This study is one of few to use the revised Professional Quality of Life scale suggested by Heritage et al. (2018) as a robust measurement alternative for assessing compassion fatigue and compassion satisfaction and preventing measurement contamination linked to the original burnout and secondary traumatic stress scales in Stamm's (2010) model. Subscale scores were calculated by taking an average of items within each subscale. Previous studies have provided support for the reliability and validity of using this version of the ProQOL‐21 (Heritage et al., 2018; Maillet & Read, 2021). In the current study, Cronbach's alpha for compassion satisfaction was 0.91 (RNs) and 0.89 (LPNs) and for compassion fatigue was 0.90 (RNs) and 0.86 (LPNs).

### 4.3 Sampling and recruitment

Ethical approval of the study was obtained from the Université de Moncton's Institutional Review Board prior to data collection (no. 1516‐071). Attendees of in‐person conferences on compassion fatigue offered by the first author in September 2019 were invited to participate in the current study by completing a self‐report survey. If attendees chose to participate, they had to read and sign an informed consent form before completing the survey. Once the survey was completed, participants were asked to deposit their completed surveys in a designated box, and their informed consent form in another. If participants chose not to participate or to withdraw their participation, they could leave their survey in a third designated box.

### 4.4 Participants

Using GPower (Faul et al., 2007), it was determined that a minimum sample size of 109 participants in each group would be required to test each of our multiple linear regression models (effect size 0.15, α = 0.05, power = 0.80). A convenience sample of 296 RNs and 110 licensed practical nurses answered a self‐administered questionnaire measuring areas of work‐life, psychological capital, emotional intelligence, compassion satisfaction and compassion fatigue. RNs and LPNs who were active members of their respective licensing boards and were attending in‐person conferences on compassion fatigue offered by the first author in four locations across the province of New Brunswick, Canada, in September 2019 were eligible to participate; attendees who were not RNs or LPNs or active members of their respective licensing boards were ineligible. Participants who met these criteria were invited to complete a self‐administered survey.

### 4.5 Data analysis

As a first step, multi‐group confirmatory factor analysis (MGCFI) was conducted in Mplus (Muthén & Muthén, 2017) to test the factor structure of each instrument and the level of measurement invariance between the LPN and RN groups among the item parameters for each validated questionnaire to ensure that group comparison was appropriate. Criteria for metric and scalar invariance was a nonsignificant chi‐square difference statistic (*p* > 0.05) as per Rutkowski and Svetina (2014). Measurement invariance between the RN and LPN groups was demonstrated for the Areas of Work‐life questionnaire, Psychological Capital Questionnaire and Emotional Intelligence Scale. Partial measurement invariance was demonstrated for the compassion satisfaction and compassion fatigue subscales of the ProQOL‐21. Descriptive statistics, reliability analyses, *t*‐tests and Pearson's *r* correlations were calculated using SPSS. Multiple linear regression in SPSS (IBM, 2012) using the forward method was used to test the effects of each area of work‐life, psychological capital and emotional intelligence on compassion satisfaction (model a) and compassion fatigue (model b). Each model was tested separately for RNs and LPNs.

## 5 RESULTS

Participant characteristics are provided in Table 1. For the RN sample, 72.0% were female (*n* = 213) while for the LPN sample, 94.5% were female (*n* = 104). For the RN sample, the largest age cohort was between the ages of 46 and 55 (*n* = 97, 32.8%), while for the LPN sample, the largest age cohort was between the ages of 36 and 45 (*n* = 39, 35.5%). The largest experience cohort for the RN sample was 31–35 years of as a nurse (*n* = 49, 16.6%) and under 5 (*n* = 27, 24.5%) for the LPN sample.

**TABLE 1:** Participant characteristics.

Variable	Registered Nurses (*n* = 296)		Licensed practical nurses (*n* = 110)	
	*N*	%	*N*	%
Gender				
Female	213	72.0	104	94.5
Male	82	27.7	5	4.5
Other	1	0.3	1	0.9
Age				
Under 25	13	4.4	9	8.2
26–35	53	17.9	18	16.4
36–45	61	20.6	39	35.5
46–55	97	32.8	26	23.6
56–65	62	20.9	18	16.4
66+	10	3.4	9	8.2
Years as nurse				
Under 5	37	12.5	27	24.5
6–10	40	13.5	24	21.8
11–15	26	8.8	25	22.7
16–20	26	8.8	15	13.6
21–25	36	12.2	8	7.3
26–30	43	14.5	5	4.5
31–35	49	16.6	2	1.8
36–40	23	7.8	3	2.7
41+	16	5.4	1	0.9

Descriptive statistics, Cronbach's alpha reliabilities and t‐test comparison results for the main study variables are presented in Table 2. There were significant differences in RNs' and LPNs' perceptions of workload, rewards, fairness at work, compassion satisfaction and compassion fatigue. RNs perceived their workload to be more manageable than LPNs (mean = 2.77, SD = 0.73 compared to mean = 2.53, SD = 0.76), perceived greater rewards (mean = 3.01, SD = 0.89 compared to mean = 2.81, SD = 0.84) and greater fairness at work (mean = 2.69, SD = 0.70 compared to mean = 2.48, SD = 0.65). Interestingly, compassion satisfaction was higher for LPNs (mean = 24.65, SD = 5.39 compared to mean = 23.43, SD = 6.16, ns), while compassion fatigue was higher for RNs (mean = 27.73, SD = 7.16) than LPNs (mean = 26.19, SD = 6.32, *p* = 0.04).

**TABLE 2:** Means, SD, Cronbach's α scale reliabilities and Independent t‐test results comparing RNs and LPNs on main study variables.

Variable	RNs (*n* = 296)			LPNs (*n* = 110)			*t*‐Value	*p*
	Mean	SD	α	Mean	SD	α		
Workload	2.77	0.73	0.59	2.53	0.76	0.73	−2.946	0.00
Control	2.92	0.88	0.82	2.92	0.81	0.75	0.001	0.99
Reward	3.01	0.89	0.81	2.81	0.84	0.76	−2.092	0.04
Community	3.29	0.80	0.81	3.19	0.72	0.81	−1.077	0.28
Fairness	2.69	0.70	0.73	2.48	0.65	0.74	−2.699	0.01
Values	3.21	0.81	0.78	3.17	0.76	0.69	−0.439	0.66
Psychological Capital	4.16	0.70	0.92	4.22	0.68	0.92	0.763	0.45
Emotional Intelligence	3.72	0.38	0.83	3.72	0.36	0.79	0.194	0.85
Compassion Satisfaction	23.43	6.16	0.91	24.65	5.40	0.89	1.822	0.07
Compassion Fatigue	27.73	7.16	0.90	26.19	6.32	0.86	−2.101	0.04

Pearson's *r* correlations between main study variables are presented in Table 3 (RNs) and Table 4 (LPNs). For RNs, all study variables were significantly correlated with compassion satisfaction and compassion fatigue. For LPNs, study variables were also significantly correlated with compassion satisfaction and compassion fatigue, except values congruence with compassion satisfaction and compassion fatigue, and workload with compassion satisfaction.

**TABLE 3:** Pearson's *r* correlations for RNs (*n* = 296).

Variable	1	2	3	4	5	6	7	8	9
1. Workload									
2. Control	0.21^a^								
3. Reward	0.33^a^	0.49^a^							
4. Community	0.17^a^	0.36^a^	0.42^a^						
5. Fairness	0.31^a^	0.50^a^	0.56^a^	0.48^a^					
6. Values	0.24^a^	0.52^a^	0.46^a^	0.38^a^	0.49^a^				
7. PsyCap	0.21^a^	0.50^a^	0.44^a^	0.36^a^	0.34^a^	0.47^a^			
8. EI	0.11	0.24^a^	0.35^a^	0.28^a^	0.18^a^	0.27^a^	0.66^a^		
9. Comp. Satisfaction	0.32^a^	0.43^a^	0.47^a^	0.25^a^	0.33^a^	0.48^a^	0.63^a^	0.45^a^	
10. Comp. Fatigue	−0.44^a^	−0.44^a^	−0.39^a^	−0.33^a^	−0.39^a^	−0.36^a^	−0.44^a^	−0.27^a^	−0.57^a^

^a^ Correlation is significant at the 0.01 level (2‐tailed).

**TABLE 4:** Pearson's *r* correlations for LPNs (*n* = 110).

	1	2	3	4	5	6	7	8	9
1. Workload									
2. Control	0.43^b^								
3. Reward	0.37^b^	0.54^b^							
4. Community	0.34^b^	0.38^b^	0.38^b^						
5. Fairness	0.33^b^	0.46^b^	0.47^b^	0.42^b^					
6. Values	0.26^b^	0.43^b^	0.41^b^	0.21^a^	0.46^b^				
7. PsyCap	0.20^a^	0.43^b^	0.44^b^	0.34^b^	0.20^a^	0.18			
8. EI	−0.04	0.14	0.22^a^	0.04	0.20^a^	0.02	0.39^b^		
9. Comp. Satisfaction	0.11	0.27^b^	0.35^b^	0.24^a^	0.20^a^	0.11	0.62^b^	0.36^b^	
10. Comp. Fatigue	−0.47^b^	−0.42^b^	−0.29^b^	−0.20^a^	−0.27^b^	−0.17	−0.39^b^	−0.21^a^	−0.37^b^

^a^ Correlation is significant at the 0.05 level (2‐tailed).

^b^ Correlation is significant at the 0.01 level (2‐tailed).

### 5.1 Compassion satisfaction

**FIGURE 2:**
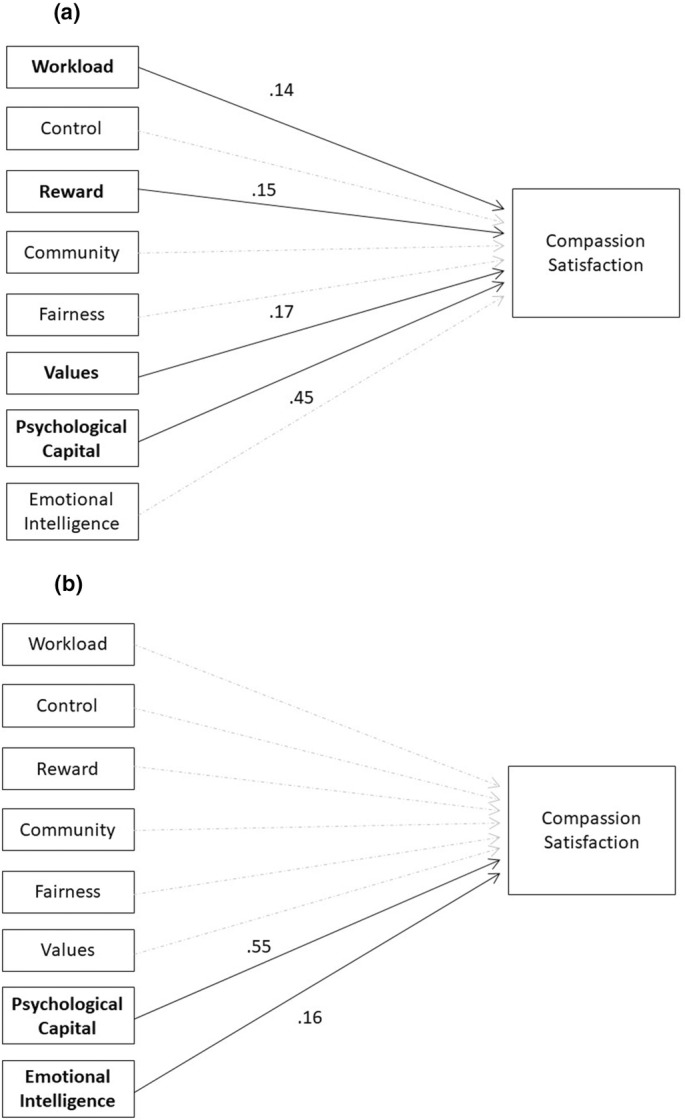
(a) Final regression model results for compassion satisfaction for RN sample. (b) Final regression model results for compassion satisfaction for LPN sample.

Psychological capital was the only variable that predicted compassion satisfaction for both RNs and LPNs. For the RN sample, the overall regression model for model a (compassion satisfaction) was statistically significant (*R* ^2^ = 0.48, *F* (4, 291) = 67.918, *p* = 0.00). For this sample, four independent variables were predictors of compassion satisfaction: psychological capital (β = 0.45, *p* = 0.000), reward (β = 0.15, *p* = 0.003), values (β = 0.17, *p* = 0.001) and workload (β = 0.14, *p* = 0.002). The final regression model results for compassion satisfaction for the RN sample is presented in Figure 2a. For the LPN sample, the overall regression model for model a (compassion satisfaction) was also statistically significant (*R* ^2^ = 0.40, *F* (2, 107) = 35.891, *p* = 0.00). Two variables, psychological capital (β = 0.55, *p* = 0.000) and emotional intelligence (β = 0.16, *p* = 0.044), significantly predicted compassion satisfaction. The final regression model results for compassion satisfaction for the LPN sample is presented in Figure 2b. Detailed regression results for model a (compassion satisfaction) are presented in Table 5 (RNs) and Table 6 (LPNs).

**TABLE 5:** Final regression model predicting Compassion Satisfaction (Model a) among RNs.

	Unstandardized coefficients		Standardized coefficients	*t*	*p*
Predictor	B	SE	β		
(Constant)	−3.647	1.718		−2.123	0.035^a^
Psychological Capital	3.980	0.436	0.454	9.124	0.000^a^
Reward	1.046	0.353	0.150	2.960	0.003^a^
Values	1.279	0.381	0.169	3.359	0.001^a^
Workload	1.181	0.379	0.140	3.116	0.002^a^

^a^ Significant at the *p* < 0.05 level.

**TABLE 6:** Final regression model predicting Compassion Satisfaction (Model a) among LPNs.

Predictor	Unstandardized coefficients		Standardized coefficients	*t*	*p*
	B	SE	β		
(Constant)	−3.672	4.548		−0.807	0.421
Psychological capital	4.407	0.640	0.554	6.890	0.000^a^
EI	0.080	0.039	0.164	2.040	0.044^a^

^a^ Significant at the *p* < 0.05 level.

### 5.2 Compassion fatigue

**FIGURE 3:**
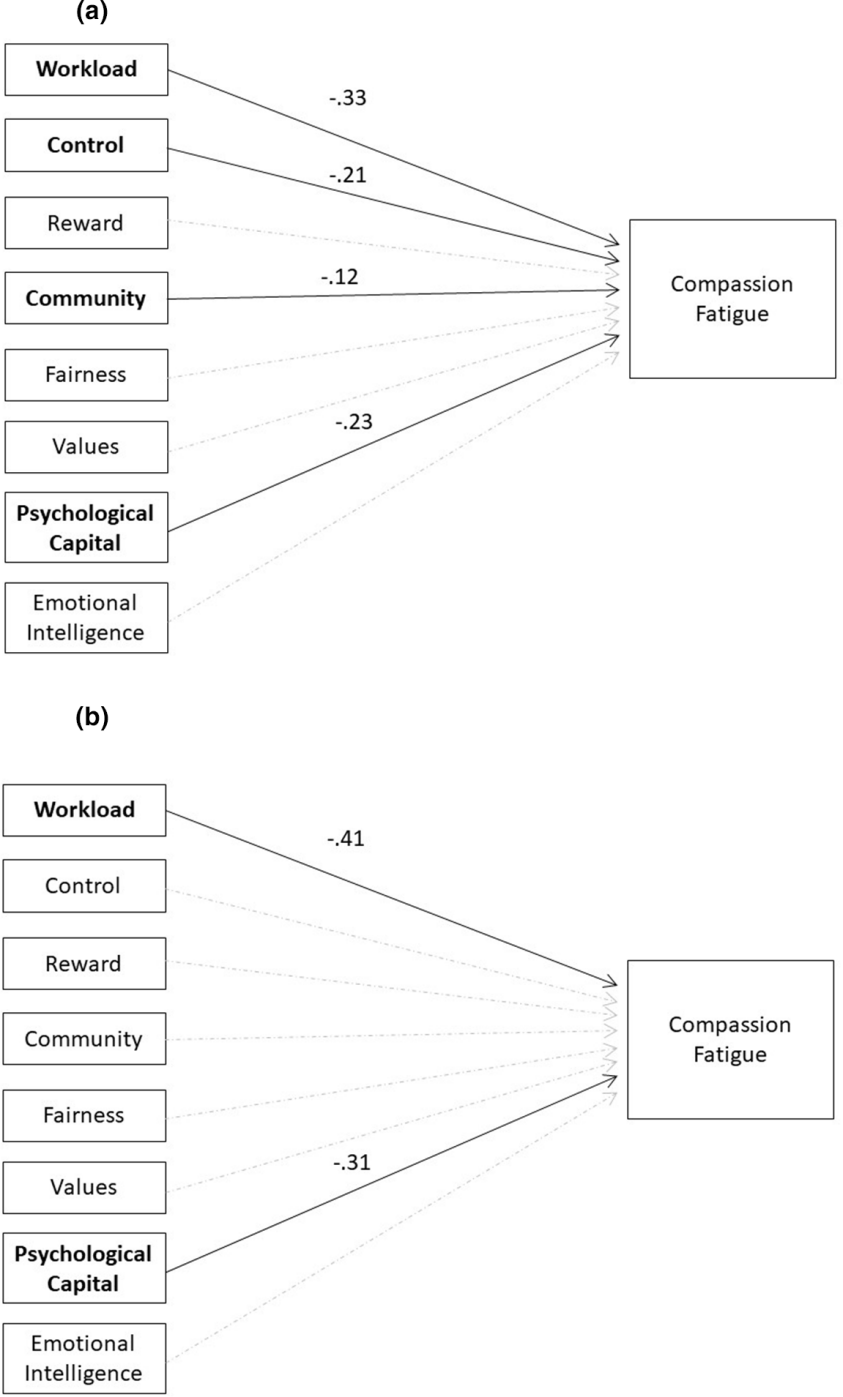
(a) Final regression model results for compassion fatigue for RN sample. (b) Final regression model results for compassion fatigue for LPN sample.

Two independent variables, workload and psychological capital, were predictors of compassion fatigue for both the RN and LPN samples. For the RN sample, the overall regression model for model b (compassion fatigue) was statistically significant (*R* ^2^ = 0.38, *F* (4, 291) = 44.278, *p* = 0.00). Four independent variables were predictors of compassion fatigue: psychological capital (β = −0.23, *p* = 0.000), workload (β = −0.33, *p* = 0.000), control (β = −0.21, *p* = 0.000) and community (β = −0.12, *p* = 0.018). The final regression model results for compassion fatigue for the RN sample is presented in Figure 3a. For the LPN sample, the overall regression model for model b (compassion fatigue) was also statistically significant (*R* ^2^ = 0.31, *F* (2, 107) = 24.202, *p* = 0.00). Two independent variables were predictors of compassion fatigue: workload (β = −0.41, *p* = 0.000) and psychological capital (β = −0.31, *p* = 0.000). The final regression model results for compassion fatigue for the LPN sample is presented in Figure 3b. Detailed regression results for model b (compassion fatigue) are presented in Table 7 (RNs) and Table 8 (LPNs).

**TABLE 7:** Final regression model predicting Compassion Fatigue (Model b) among RNs.

Predictor	Unstandardized coefficients		Standardized coefficients	*t*	*p*
	B	SE	β		
(Constant)	54.883	2.257		24.320	0.000^a^
Psychological Capital	−2.318	0.561	−0.227	−4.134	0.000^a^
Workload	−3.234	0.470	−0.329	−6.887	0.000^a^
Control	−1.722	0.447	−0.211	−3.850	0.000^a^
Community	−1.076	0.454	−0.121	−2.371	0.018^a^

^a^ Significant at the *p* < 0.05 level.

**TABLE 8:** Final regression model predicting Compassion Fatigue (Model b) among LPNs.

Predictor	Unstandardized coefficients		Standardized coefficients	*t*	*p*
	B	SE	β		
(Constant)	46.919	3.365		13.944	0.000^a^
Workload	−3.382	0.684	−0.405	−4.944	0.000^a^
Psychological capital	−2.889	0.762	−0.311	−3.790	0.000^a^

^a^ Significant at the *p* < 0.05 level.

## 6 DISCUSSION

The purpose of this study was to investigate the impact of six areas of work‐life (workload, control, reward, fairness, community, values congruence), emotional intelligence, and psychological capital on compassion fatigue and compassion satisfaction among RNs and LPNs. To our knowledge, this is the first study to examine these variables concomitantly. This research also addresses a gap in the nursing literature by investigating the understudied LPN nursing population and comparing it to RNs.

In our hypothesized models, we proposed that RNs and LPNs who perceived a greater degree of person‐job fit in the six areas of work‐life, higher emotional intelligence and greater psychological capital would experience higher levels of compassion satisfaction and lower levels of compassion fatigue. These hypothesized relationships were partially supported.

As expected, all study variables were significantly correlated with compassion satisfaction and compassion fatigue for RNs. For LPNs, most study variables were also significantly correlated with compassion satisfaction and compassion fatigue, except for values congruence with compassion satisfaction and compassion fatigue, and workload with compassion satisfaction. Overall, these results suggest that multiple areas of work‐life, and emotional intelligence and psychological capital, have a significant association with nurses' compassion satisfaction and compassion fatigue. Additionally, these relationships appear to differ somewhat between RNs and LPNs.

The regression results for the RN sample found that positive psychological capital, a feeling of being recognized and rewarded for their work, an alignment between personal and organizational values, and a manageable workload significantly predicted compassion satisfaction. Similarly, psychological capital was a significant predictor of compassion satisfaction for LPNs in our study. However, emotional intelligence was also an important antecedent for LPNs, but not for RNs, and none of the areas of work life were significant predictors of compassion satisfaction for this group of nurses.

In terms of compassion fatigue, psychological capital, workload, control and community significantly predicted lower levels of compassion fatigue among RNs, whereas only psychological capital and workload were significant predictors of this outcome among LPNs. Psychological capital was a significant predictor variable in all four regression models. Thus, the findings highlight the primary importance of psychological capital as an internal personal resource influencing both compassion satisfaction and compassion fatigue among RNs and LPNs. RNs and LPNs may benefit significantly from greater psychological capital, possibly due to heightened abilities to effectively cope with adversity and respond to challenging situations with hope, efficacy, resilience and optimism (HERO) (Luthans et al., 2004; Read & Laschinger, 2013). So‐called “HERO” nurses are better equipped to identify, meet and solve patients' complex care needs. They may be more confident in their ability to cope with workplace challenges, highly motivated to suggest innovative solutions and able to recover from adversity more quickly. They may also view stressful situations more positively and adapt more effectively to stressors present in their work environments (Yao et al., 2022; Yildrim et al., 2021). Few previous studies have addressed the relationships between psychological capital and compassion fatigue and compassion satisfaction among RNs. However, encouraging results were yielded in a study conducted by Bao and Taliaferro (2015) which found that psychological capital was negatively correlated with compassion fatigue and positively correlated with compassion satisfaction among acute care RNs. More recently, Yildrim et al. (2021) also found psychological capital to have a direct but weak effect on RNs' compassion fatigue, while a strong positive relationship was found between psychological capital and compassion satisfaction. We are the first to report on these relationships among LPNs.

Consistent with previous research, our results show that areas of work life have an important influence on nurses' compassion satisfaction and compassion fatigue. Specifically, our results showed that workload, rewards, control, values congruence, and a sense of community are particularly important, although there were differences between the two groups.

Previous studies of RNs have demonstrated that when RNs report feeling rewarded and recognized for their work, an alignment between their personal values and the organization's values and that their workload is manageable, they have higher levels of compassion satisfaction (Boamah & Laschinger, 2016; Harr, 2013; Killian, 2008; Slatten et al., 2011). Our findings corroborate existing literature, which indicates that when nurses perceive their workloads to be unmanageable, they experience time pressure to accomplish their work as quickly as possible, leaving inadequate time to provide optimal, holistic, patient‐centred care and are, therefore, more likely to experience compassion fatigue (Boamah & Laschinger, 2016; Towey‐Swift & Whittington, 2021). For example, in a study conducted by Towey‐Swift and Whittington (2021) among community mental health teams, an unmanageable workload was significantly related to compassion fatigue. In the same study, only workload was consistently predictive across compassion fatigue and compassion satisfaction, which corroborates our study findings on the importance of workload as an influential work environment characteristic that contributes to how nurses' experience their work. This is not surprising since compassion fatigue describes a type of physical, emotional and psychological exhaustion resulting from chronic work‐related stress exposure, including excessive workloads and an inability to control patient outcomes (Peacock, 2023; Xie et al., 2021). In another study conducted by Boamah and Laschinger (2016), a sense of community in the workplace was found to be crucial as it fosters a greater sense of trust and cohesiveness and enables effective communication, shared responsibility, and feeling valued as a member of such a community. Since nurses may draw upon their social support systems to counteract the negative effects of work demands and work stress, a poor sense of community could understandably be linked to higher levels of compassion fatigue. Contrary to RN results, workload, rewards and values were not predictive of compassion satisfaction for LPNs, nor were control and sense of community predictive of compassion fatigue. These findings were surprising, given the overlap in the role and scope of practice of LPNs and RNs. However, on average LPNs reported higher workloads, higher levels of compassion satisfaction and lower levels of compassion fatigue than the RNs in our study, which may suggest that these two groups of nurses experience their work differently. This may partially explain why the model results were not identical for both groups. To our knowledge there are no other published studies reporting these relationships among LPNs, so we are unable to compare our results to previous research.

Our study findings suggest that emotional intelligence is a significant predictor of compassion satisfaction among LPNs, but not for RNs. Therefore, LPNs ability to understand and regulate their own feelings while being empathetic to others is an important predictor of their compassion satisfaction. Surprisingly, emotional intelligence was not a predictor of either compassion satisfaction or compassion fatigue for the RN group. In a recent study conducted by Maillet and Read (2021), comparable results among a sample of 1271 nurses were found. The researchers argued that nurses' compassion satisfaction and compassion fatigue may be impacted by the emotional dissonance that is created when there is a mismatch between the emotions nurse's experience and the socially acceptable emotions they must display in caregiving contexts, rather than the nurses' degree of emotional intelligence. For instance, some nurses may adapt to expected emotional reactions during patient care by using emotional labour rather than emotional intelligence (Lu & Shorey, 2021; Maillet & Read, 2021). Because of the frenetic pace and emotionally demanding nursing work environment, we further argue that nurses may not have time to process, understand, and manage their emotions adequately. In support of this explanation, Yesil and Polat (2023) argued that nurses use problem‐focused coping styles, rather than emotion‐focused, in stressful situations. More specifically, nurses first try to cope with stressful situations by changing the source of stress before regulating their emotions. In line with this, Lu and Shorey (2021) suggested that emotional intelligence can be displayed inconsistently in the nursing context due to prevailing circumstances, such as a hectic clinical setting, time constraints, poor staffing levels, scarce resources and increasing workloads. When the emotional energy dispensed by nurses in their efforts to provide compassionate care in stressful working settings exceeds their emotional capabilities, nurses can dip into other resources to restore their emotional reservoir (Al‐Marri et al., 2020; Vilariño del Castillo & Lopez‐zafra, 2022; Yildrim et al., 2021), such as psychological capital.

Another interesting finding in this study was the significant difference in RNs' and LPNs' perceptions of workload, rewards, fairness at work and both compassion satisfaction and compassion fatigue. RNs perceived their workload to be more manageable than LPNs and perceived greater rewards and greater fairness at work. Interestingly, compassion fatigue was higher for RNs than LPNs while compassion satisfaction was higher for LPNs than RNs. These differences might be explained by the different scopes of practice and the unique work characteristics of RNs and LPNs. While the complementary nature of LPNs' and RNs' scopes of practice requires a high level of interprofessional collaboration (Montayre & Montayre, 2017; Squires et al., 2019; Thompson et al., 2021), RNs take on greater levels of responsibility and clinical decision making, requiring more education and training, and as a result receive higher pay than LPNs (Thompson et al., 2021). Moreover, LPNs and RNs across Canada also typically belong to separate unions with different rules for seniority, overtime pay, pensions, etc. so their “rewards” are quite different from this perspective. RNs also often take on supervisory or leadership roles, which may contribute to explaining why RNs perceive their workload to be more manageable and perceive greater rewards and fairness at work than LPNs. Furthermore, RNs are assigned more complex patient cases and are required to engage in higher levels of critical thinking and clinical judgement than LPNs (Thompson et al., 2021), therefore, potentially contributing to higher levels of compassion fatigue and reduced compassion satisfaction. LPNs are assigned more stable and predictable patient cases and dispense direct patient care based on the care plans established by RNs (Canadian Institute for Health Information, 2021; Phillips et al., 2021). Since LPNs may spend more time in direct patient care with less acute patient cases than RNs (Montayre & Montayre, 2017; Thompson et al., 2021), they may feel a broader sense of gratification from caregiving (Jakimowicz et al., 2018), which could explain why LPNs reveal higher compassion satisfaction than RNs in the current study.

### 6.1 Future research and limitations

Our study is the first to examine the impact of areas of work‐life, psychological capital and emotional intelligence on compassion fatigue and compassion satisfaction among LPNs, along with a comparison of these relationships among RNs. The findings contribute to the nursing literature by showing that psychological capital is a valuable intrapersonal resource for both groups of nurses and that different areas of work‐life impact compassion satisfaction and compassion fatigue for RNs and LPNs. Future studies should investigate the nature and span of these differences to better understand differences in the work experiences of RNs and LPNs. It would also be interesting to investigate whether RNs consistently report higher compassion fatigue than LPNs across other samples. Researchers could also further investigate the reasons why LPNs have lower positive perceptions of their workload, rewards and fairness at work than their RN counterparts. The lack of significant findings beyond psychological capital on both compassion fatigue and compassion satisfaction among LPNs in this study raises questions about their antecedents among this group of nurses. However, due to our small convenience sample, further research is necessary to corroborate our results.

Some drawbacks of this study should be acknowledged. First, the choice of a cross‐sectional research design prevented our ability to draw conclusions about causality and directionality. Therefore, longitudinal studies to test causal inferences between variables are warranted in the future. Second, there is potential for a response bias and common method variance as a result of using multiple self‐report surveys in a single study. It is also possible that there are additional variables that were not included in our study that account for variance in compassion fatigue and compassion satisfaction. Third, although the sample sizes had adequate power to test the hypothesized models, its generalizability is limited. Indeed, the small convenience sample of this study limits the potential representativeness of our study results, especially among the understudied LPN nursing population. In future studies, researchers are encouraged to broaden the research around LPNs with larger samples.

### 6.2 Implications for practice

These study findings have several key implications for practice. First, multi‐level interventions including engagement from policymakers, administrators, nursing leaders and front‐line nurses are warranted to identify modifiable work environment factors and collectively devise strategies to promote compassion satisfaction and prevent compassion fatigue by creating and maintaining work environments where both RNs and LPNs can thrive. According to Towey‐Swift and Whittington (2021), nursing workloads are unlikely to be significantly reduced anytime soon due to the context of workforce shortages, limited resources and complexity of patient care. Therefore, it is important to consider approaches that attenuate nurses' workload and efforts to improve psychological capital among both RNs and LPNs. A strengths‐based approach centred on fostering nurses' psychological capital through ongoing training and intervention actions could be beneficial for both RNs and LPNs. These strategies should be implemented and tested to directly assess their impact on both compassion fatigue and compassion satisfaction among RNs and LPNs. Our results could potentially inform new initiatives aimed at further supporting LPNs from an organizational perspective.

## 7 CONCLUSIONS

This study identified several areas of work‐life and intrapersonal resources associated with compassion satisfaction and compassion fatigue among RNs and LPNs. Knowledge of these predictors provides key direction for reducing the prevalence of compassion fatigue and increasing the prevalence of compassion satisfaction. The most compelling predictor identified in this study was psychological capital. Investments in the development and improvement of nurses' psychological capital should be prioritized since it is a malleable and impactful intrapersonal resource on compassion satisfaction and compassion fatigue. The results of this study also underscore the need to create work environments for RNs and LPNs that foster a manageable workload and positive relationships, where nurses have professional autonomy, decision‐making capacities, and access to adequate resources to do their job effectively. The nursing work environment should also allow recognition of nurses' contribution and an alignment between personal and organizational values. Such structural empowerment is a key component of healthy nursing work environments, which highlights the importance of shaping the context of the work environment.

## AUTHOR CONTRIBUTION

**Stephanie Maillet:** Conceptualization (lead); investigation (lead); project administration (lead); writing—original draft (lead); review and editing (equal). **Emily A. Read:** Methodology (lead); Formal statistical analysis (lead – descriptive, correlational, regression); writing—review and editing (equal).

## FUNDING INFORMATION

This research received no specific grant from any funding agency in the public, commercial or not‐for‐profit sectors.

## CONFLICT OF INTEREST STATEMENT

The authors declare no conflict of interest with respect to the research, authorship and/or publication of this article.

## Data Availability

Research data not available due to confidentiality of data.
